# Genetic diversity of the human immunodeficiency virus of type 1 in Gabonese transfusional settings

**DOI:** 10.1186/s12879-023-08154-7

**Published:** 2023-03-30

**Authors:** Christian Mangala, Joseph Fokam, Denis Maulot-Bangola, Olivier Rebienot-Pellegrin, Thérèse Nkoa

**Affiliations:** 1 Catholic University of Central Africa (CUCA), Yaoundé, Cameroon; 2 National Public Health Laboratory (NPHL), Libreville, Gabon; 3 Chantal Biya International Reference Center (CBIRC)), Yaoundé, Cameroon; 4 National Blood Transfusion Center (NBTC), Libreville, Gabon

**Keywords:** HIV-1, Genetic diversity, Residual risk, NBTC of Gabon

## Abstract

**Background:**

The high endemicity of transfusion-transmissible infections (TTIs) in sub-Saharan Africa is a real public health problem. To reduce the risk of HIV transmission through blood donation, the NBTC of Gabon has launched in recent years a reorganization of its blood transfusion system. This study aims to characterize the molecular strains of HIV-1 circulating in donors and to estimate the risk of viral transmission.

**Materials and methods:**

A cross-sectional study was carried out during the period from August 2020 to August 2021 among 381 donors who had agreed to donate blood at the National Blood Transfusion Center (NBTC). Viral load was determined by Abbott Real-Time (Abbott m2000®, Abbott) and sequencing by the Sanger method (ABI 3500 Hitachi®). The phylogenetic tree was constructed by MEGA X software. Data were checked, entered, and analyzed using SPSS version 21.0 software, with *p* ≤ 0.05 considered statistically significant.

**Results:**

A total of 381 donors were enrolled in the study. Among the 359 seronegative donors, five (5) seronegative donors were detected positive for HIV-1 using Real-Time PCR. The residual risk was 648 per 1,000,000 donations. The prevalence of residual infection was 1.4% [0,01; 0,03]. Sixteen (16) samples were sequenced. The strains obtained were CRF02_AG (50%), subtype A1 (18.8%), subtype G (12.5%), CRF45_cpx (12.5%) and subtype F2 (6.2%). Six sequences clustered with A1, G, CRF02_AG, and CRF45_cpx subtypes.

**Conclusion:**

The residual risk of HIV-1 transmission by blood transfusion remains a concern in the Gabonese transfusional settings. A policy based on improving the current screening strategy would involve the implementation of the nucleic acid test (NAT) in order to optimize the safety of the donation by detecting the HIV-1 subtypes in circulation in the donors.

## Background

The residual risk of HIV is a problem that daily threatens the safety of donation in all blood banks around the world. The transmission of transfusion-transmissible infections (TTIs) remains a real problem in transfusion settings around the world [1–5].

In developed countries, this risk has been reduced considerably for more than a decade in the transfusion settings of these countries. The financial means granted by these countries have contributed to the improvement of transfusion safety in order to prevent any transmission of infectious agents [6–9].

Blood transfusion safety in developing countries is constantly threatened by bloodborne pathogens circulating in the population. These pathogens are essentially transmitted during the serological window (i.e. the time when the infection is transmitted and the time when the serological test can reliably detect this infection). However, the screening strategies put in place to secure blood donations still prove to be ineffective in considerably reducing the residual risk of infectious agents (such as HIV) in blood banks. These countries must then review their strategy about transfusion safety in the various blood banks [10–14].

In Gabon, the risk of HIV transmission was estimated at 64.7 per 1,000,000 donations in 2014. The safety of the donation must be reinforced considerably by better-adapted screening strategies. However, means are needed to achieve optimal donation security. The quality of reagents used in blood banks needs to be reviewed. Ensure that these reagents have been pre-screened locally to ensure the safe screening of blood donations. The evaluation should not be limited to rapid screening tests (RDTs) but also to 4th generation tests (ELISA) dedicated to blood donation screening. If these conditions are not respected, this can weaken the security of the donation by exposing the recipient to any infectious intrusion [15–17].

The inefficiency of certain Elisa tests to be able to ensure the safety of the donation must require the implementation of nucleic acid research by the use of the nucleic acid test (NAT). This will considerably reduce the risk of transmission of viral strains in a transfusion setting. This is explained by the difference in the two residual risks estimated at the NBTC in 2014 and during the study (in 2021), which is probably due to the techniques (ELISA and NAT respectively) used for the detection of incident cases. This is why countries in Sub-Saharan Africa must implement NAT in the largest banks in the countries [18–21].

The strains of HIV-1 circulating in the population are significant. This genetic diversity of HIV-1 which is important in Gabon is justified by its geographical location because Gabon is one of the countries of Central Africa which abounds in almost all strains of HIV-1 [22]. Detailed knowledge of current genetic diversity allows better care of infected people and monitoring of strains in the evolution of the infection. Controlling genetic diversity in transfusional setting also makes it possible to better strengthen donation screening strategies because this genetic diversity has a negative impact on serological screening tests (on the sensitivity and accuracy of the test) increasing the risk of transmission.

The objective of this study is to estimate the risk of viral transmission and to characterize the molecular strains of HIV-1 circulating in donors.

## Methods

### Study design and setting

A cross-sectional study was conducted at the National Blood Transfusion Center (NBTC) in Gabon and at the Chantal Biya International Reference Center (CBIRC) in Cameroon during the period from August 2020 to August 2021. The serological analysis (ELISA 4th generation) was carried out at the NBTC and the molecular analysis (viral load and sequencing) was carried out at the CBIRC. The donors included in the study were eligible for the study criteria (being between the ages of 18 and 55, Having donated blood to the NBTC only, etc.) and having donated blood once during the collection period for the estimation of the residual risk. of HIV-1.

### HIV serological screening algorithm in a transfusion setting

**Fig. 1:**
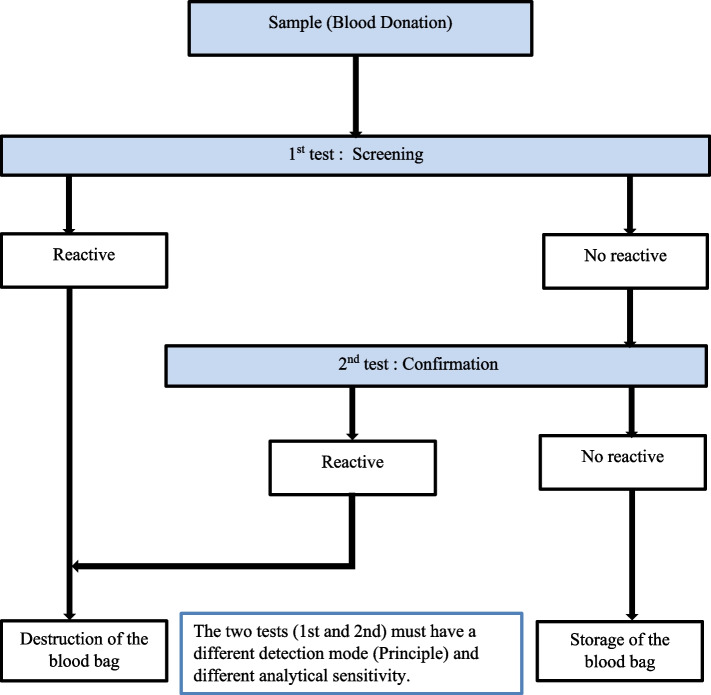
Algorithm for the diagnosis of HIV in a transfusion setting

The implementation and rigorous application of a screening algorithm have always contributed to the safety of donation in transfusion setting. The safety of blood donation has always been part of the field of biological qualification. The screening of HIV in a transfusion setting was carried out taking into account an algorithm allowing better discrimination of HIV in blood banks, particularly in countries with limited resources [23]. For this, certain serological markers such as the anti-HIV-1/2 antibody and the p24 antigen have always been sought in blood donors in the context of limited resources. And for this, two serological tests were used simultaneously (ELISA 4th generation and Chemiluminescence: case of the NBTC). The first test was used to screen all donations and the second test confirmed all donations declared negative on the first test. Only blood bags declared negative on both tests (1st test and 2nd test) were used for distribution to recipients. On the other hand, all blood bags with positive serology at the first test and indeterminate serology were destroyed (Fig. 1).

### Serological analysis

A blood sample was taken in an EDTA tube for serological analysis from each donor. The techniques used to detect the p24 antigen and anti-HIV-1 (groups M and O) and HIV-2 antibodies in donor plasma were the ELISA technique (Evolis®, BioRad, France), and the chemiluminescence technique (Cobas® 6000 e601, Roche, Germany). Serological analysis was performed according to the manufacturer's protocol.

### Molecular analysis

#### Viral load quantification

Quantification of the viral load was carried out using the Real-Time PCR technique (Abbott m2000®, Abbott, USA) by searching for nucleic acid (RNA) in the plasma of donors where the extractive phase had taken place on Abbott m2000sp and the amplification phase on Abbott m2000rt. Molecular analysis was performed according to the protocol of the manufacturer Abbott. The molecular analyzes were carried out at the CBIRC. The different primers used for amplification:

- Prime direction: 5'GAC AGG CTA ATT TTT TAG GG 3',
- Antisense primer: 5' TTT CCC CAT ATT ACT ATG CTT 3'.

#### Sequencing of HIV-1 strains

All the sixteen strains sequenced were selected after having analyzed all the seropositives by Real-time PCR. Depending on the sequencing technique used (Sanger method, ABI 3500, Hitachi, Japon), the detection of viral genotypes/strains would be limited to majority populations (covering at least 20% of the viral population present in the individual concerned). A nested PCR was performed to separately amplify the protease (PR) and reverse transcriptase (RT) genes from the cDNA synthesized using the Invitrogen® kit. The reaction mixture consisted of 0.75 µl of the BS primer, 0.75 µl of the TAK3 primer, 0.8 µl of dNTPs, 3 µl of MgCl2, 5 µl of Buffer TAQ 10x, 33.95 µl of H2O, and 0 0.75 µl of TAQ Gold. Then 45 µl of the master mix was dispensed into the microtube while working on ice. Then 5 µl of the sample was distributed in the microtube under the hood. And the amplification was done in a thermal cycler. A fragment of amplicons for the PR and RT genes were generated and confirmed by agarose gel electrophoresis. The purification of the Nested-PCR product was done by adding 5 µl of enzymes (ExoSAP-IT) in 13 µl of the Nested-PCR product and it was carried out in the thermal cycler. The sequencing reaction used eight (8) primers (B, F, SEQ1, SEQ2, SEQ3, SEQ4, SEQ5, and TAK3) which completely covered the fragment (about 1300 Bp) to be sequenced. The reaction mixture was composed of 3.2 µl of primers, 1.5 µl of Big Dye, 6.5 µl of Big Dye diluent, and 4.8 µl of H2O. An optical plate was used by distributing in each well 12 μl of Formamide HiDi then 7 μl of the purified sequence reaction. The denaturation is carried out at 95° C. for 2 min. The reading of the optical plate is done using the ABI 3500 Hitachi sequencer. The RECALL® program was used for the interpretation of the chromatogram. The sequences were exported to Stanford HIV data for the identification of the strains involved.

The primers for the amplification of the Nested PCR were:

- 5' GAC AGG CTA ATT TTT TAG GG 3'
- 5' GGC TCT TGA TAA ATT TGA TAT GT 3',

The primers used for the sequencing reaction were:

- 5’ AGC AGA CCA GAG CCA ACA GC 3’
- 5’ CCA TCC ATT CCT GGC TTT AAT 3’
- 5’ CAG GAA TGG ATG GCC CAA AA 3’
- 5’ TTG TAC AGA AAT GGA AAA GGA AGG 3’
- 5’ CCC TGT GGA AAG CAC ATT GTA 3’
- 5’ GCT TCC ACA GGG ATG GAA A 3’
- 5’ CTA TTA AGT CTT TTG ATG GGT CA 3’
- 5'-TTT CCC CAT ATT ACT ATG CTT-3'

### Phylogenetic analysis

The evolutionary history of the strains was inferred using the Neighbor-Joining method. Phylogenetic analysis was performed after obtaining the sequences. The sequences were downloaded from the Los Alamos database. All sequences were purified and aligned using BioEdit software. The construction of the phylogenetic tree of reverse transcriptase (RT) and protease (PR) sequences using Neighbor joining was generated by MEGA X software [24, 25]. The bootstrap consensus tree inferred from 500 replicates was taken to represent the evolutionary history of the HIV-1 strains analyzed. Evolutionary distances were calculated using the composite maximum likelihood method and are expressed as the number of base substitutions per site.

### Statistical analysis

Data were checked, entered, and analyzed using SPSS software version 21.0 and EPI info 7.0. Descriptive data were presented as frequencies and percentages. The incidence rate (IR) was calculated for donors who were HIV-negative at baseline and became virus-positive (viral load detectable) after analysis by Real-Time PCR as the number of incident cases divided by the total number of person-years (PY). The number of person-years was calculated by multiplying the total study population by the study duration. The residual risk (RR) of transmission of a viral infection linked to the window period was estimated from the Schreiber equation by multiplying the incidence rate by the duration of the window period (17 days due to the technique used which is ELISA but 22 days relate to RDTs) and the whole divided by 365 [26, 27]. The *P*˂0.05 value was considered statistically significant.

## Results

### Prevalence of HIV-1 infection according to socio-demographic data, *N* = 381

A total of 381 donors were enrolled during the study. The prevalence of HIV-1 had no significant difference between men (7%) and women (7.2%), with *P* = *0.9482*. The age group that had a slightly high prevalence was between 18 and 28 years old, which was 11.9% but not significant. A high prevalence of 13.2% was found in new donors, which was significantly higher than in regular donors (3%), *P* = *0.0002* (Table 1).

**Table 1:** Prevalence of HIV infection according to socio-demographic data, *N* = 381

Variables		Neg (%)	Pos (%)	***P***-value
Sex	Male	238 (93	18(7)	0.9482
	Female	116(92.8)	9(7.2)	
Age (years)	18–28	59 (88.1)	8 (11.9)	0.1437
	29–39	202 (93.1)	15 (6.9)	
	40–50	69 (94.5)	4 (5.5)	0.5691
	> 51	24	0	
Donation statua	Former/Regular	223 (97)	7 (3)	0.0002
	New	131 (86.8)	20 (13.2)	
Type of donor	Unrelated Voluntee	79 (91.9)	7 (8.1)	0.6391
	Family	275 (93.2)	20 (6.8)	

*Neg* Negative, *Pos* Positive, *%* Percentage, *N* Number

### Risk of HIV transmission in transfusional setting

During the study, 381 donors were included in the study, ie 359 seronegative and 22 seropositive. Out of 359 seronegative, 5 seronegative donors proved to be viropositive (positive viremia) by Real-time PCR. The incidence rate (IR) and the residual risk (RR) were estimated, i.e. 1,400 per 100,000 person-years (PY) and 648 per 1,000,000 donations respectively. The prevalence of residual infection was 1.4% [0,01; 0,03] (Table 2).

**Table 2:** Estimation of residual risk and prevalence of residual infection in NBTC donors

Person-Year	Residual case	PRI [CI]	IR for 100,000 PY	RR for 1,000, 000 of donations
359	5	1.4% [0.01; 0.03]	1,400	648

*PY* Person-Year, *PRI* Prevalence of Residual Infection, *IR* Incidence Rate, *RR* Residual Risk, *CI* Confidence Interval

### Characterization of circulating HIV-1 strains in donors

**Fig. 2:**
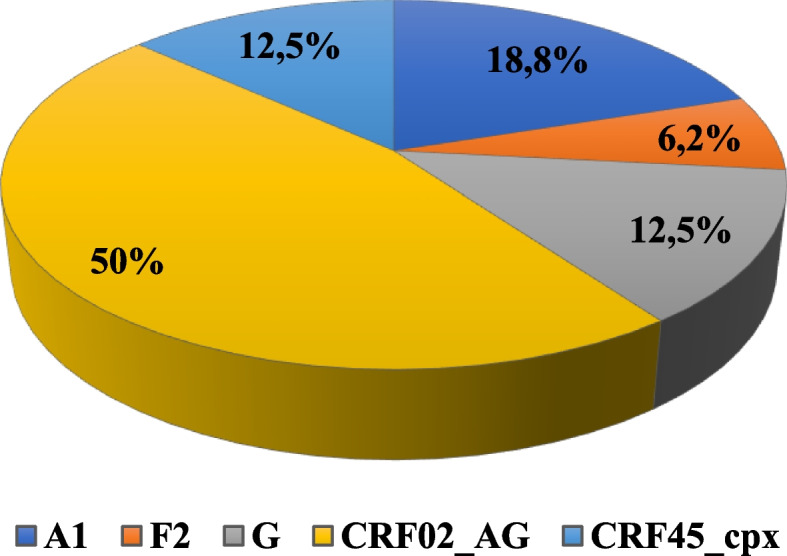
Characterization of molecular strains of HIV-1 in donors

The study made it possible to characterize the strains of HIV-1 circulating in the donors and also to determine their frequency of appearance. During the study, 16 strains of HIV-1 were sequenced. And out of 16 strains, there were 3 (18.8%) for the A1 subtype, 1 (6.2%) for the F2 subtype, 2 (12.5%) for the G subtype, 2 (12.5%) for CRF45_cpx, and 8 (50%) for CRF02_AG (Fig. 2).

### Molecular phylogeny of HIV-1 strains

**Fig. 3:**
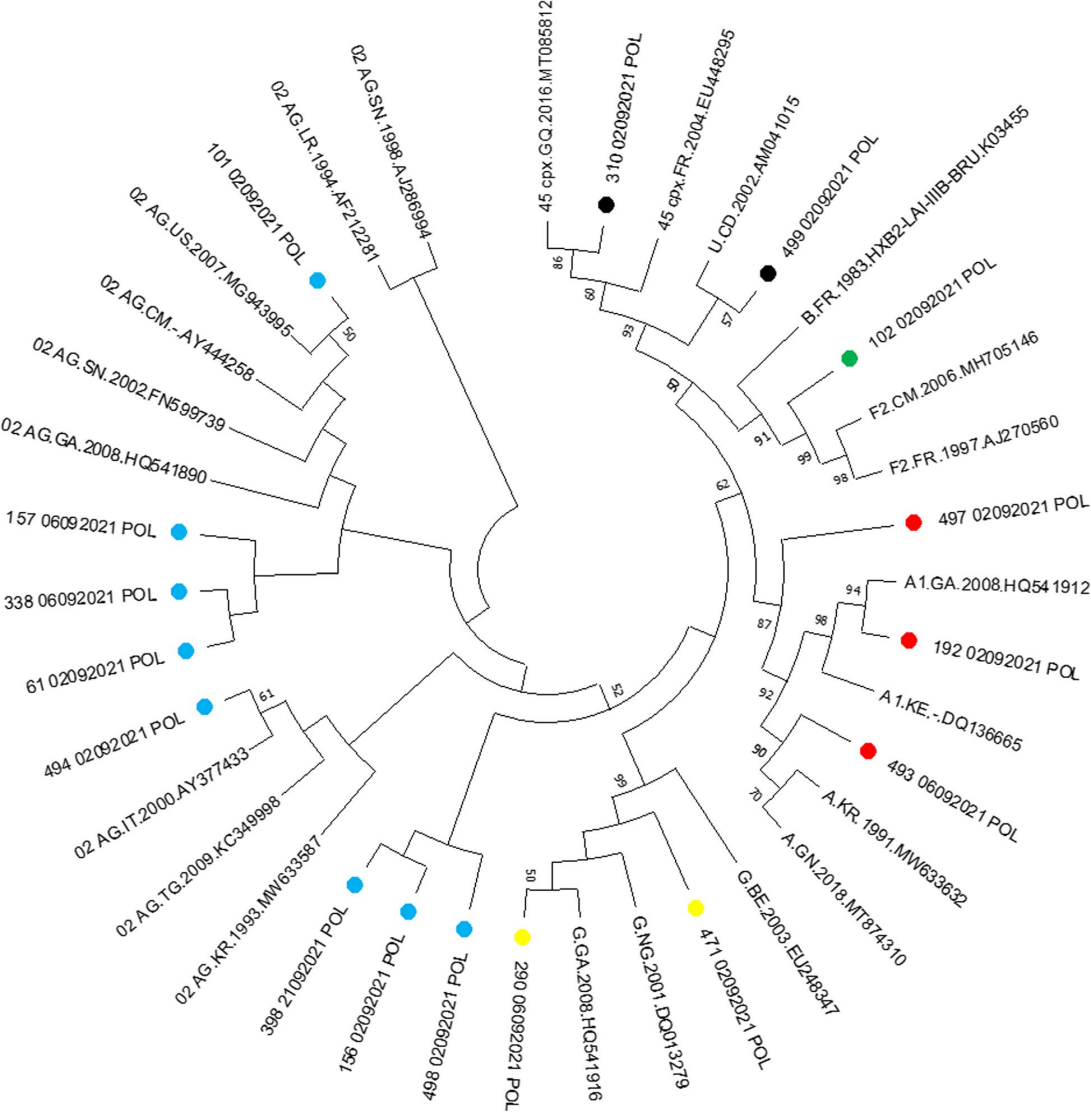
Phylogenetic tree of HIV-1 strains of interest and reference strains downloaded from the Los Alamos database. Out of eight identified clusters, two clusters are composed of sequences of interest which have grouped together and in the other six clusters it is the sequences of interest which are grouped around the reference sequences identified in Gabon, Equatorial Guinea, Italy, the Democratic Republic of Congo and the United States. A1, F2, G, CRF02_AG and CRF45_cpx strains were identified with colored symbols in red, green, yellow, blue and black respectively. Branches corresponding to partitions replicated in less than 50% of bootstrap replicates have been collapsed. The percentage of replicated trees in which related strains are clustered in the bootstrap test (500 replicates) is shown next to the branches

Phylogenetic analysis of reverse transcriptase (RT) and protease (PR) sequences was performed with the Molecular Evolutionary Genetic Analysis Tool version 10.0 (MEGA X). There was an evolutionary relationship between HIV-1 strains. The 10102092021POL and 49402092021POL sequences clustered with the recombinant CRF02_AG. The 31002092021POL and 49902092021POL sequences clustered with the recombinant CRF45_cpx. The 19202092021POL sequence clustered with the subtype A1 and the 29006092021POL sequence clustered with the subtype G (Fig. 3).

## Discussion

The residual risk of HIV-1 transmission in a transfusion environment is still a current problem in blood banks in all developing countries in general and in Gabon in particular. Our study looked at the residual risk of HIV-1 transmission and the genetic diversity of HIV-1 in blood donors from the National Blood Transfusion Center of Gabon. The study revealed that the prevalence of HIV-1 had no significant difference between men (7%) and women (7.2%), *P* = 0.9482. However, the prevalence (13.2%) was significantly higher (*P* = 0.0002) in new donors than in regular donors. It is important to note that according to the WHO, new donors are at greater risk. In addition, new donors most often visit blood banks for the sake of knowing their HIV status for free after observing risky behavior. Some studies in Pakistan, Cameroon, Netherlands, Central African Republic (CAR), and Nigeria have also shown that new donors have high positivity [28–32]. Indeed, the prevalence of residual HIV-1 infection was 1.4%. But also the residual risk of HIV-1 transmission in the Gabonese transfusional settings, more precisely at the National Blood Transfusion Center in Libreville, was estimated at 648 per 1,000,000 donations as well as the incidence rate which was 14 per 1,000 people- years. These results showed that the residual risk of HIV-1 at the NBTC in Libreville was high compared to that estimated in 2014, which was 64.7 per 1,000,000 donations. And this could be explained by an inefficient screening strategy despite the use of 4th generation screening tests which sometimes do not undergo local evaluation or by the absence of tests detecting viral nucleic acid (RNA or DNA). in plasma. But also these data could be due to the different donor selection strategies implemented in blood banks that almost do not meet the requirements dictated by the World Health Organization (WHO) in order to eliminate a large acceptable number of donors. at risk. Some studies conducted in Cameroon, Ghana, Burkina Faso and Tanzania have shown that the residual risk of HIV-1 is present and high in several blood banks in resource-limited countries [15, 33–37]. It should be noted that certain studies carried out in several countries have shown that the screening strategy based on the detection of antibodies and p24 antigen for HIV was limited compared to detection based on RNA research. This observation would become a necessity for the implementation of the nucleic acid test (NAT), especially in large transfusion centers in countries with limited resources. And it is preferable for the detection of infectious agents in blood banks to use a NAT with a lower detection threshold (for example 13.2 copies/ml) [38–41]. HIV-1 strains circulate daily in blood banks, more precisely at the NBTC in Gabon. The study had identified molecular strains that circulate among NBTC blood donors. And the frequency of appearance of these different strains was higher for CRF02_AG, for the A1 subtype, for the G subtype and for CRF45_cpx. The frequency of circulation of these strains of HIV-1 among blood donors could be justified by the geographical location of the country which is one of the countries of Central Africa where almost all the different strains of HIV are found. -1. But also certain activities such as tourism and travel have allowed the circulation of these strains from one country to another, from one continent to another. Some studies conducted in several countries of the world namely in Ghana, Nigeria, Cameroon and Mozambique have shown that the recombinant forms CRF02_AG and the A1 subtype were more representative [42–45]. Previous studies conducted in Gabon have shown an increasing genetic diversity of HIV-1 circulating in the general population. This growth is in favor of CRF02_AG strains (46.7%), subtypes A (19.6%), G (10.3%), F (4.7%), H (1.9%) and D (1.9%) [46]. The data from this study confirms, despite the small size of the study, that the CRF02_AG recombinants are in the majority, followed by the A1, G, F2 and CRF45_cpx strains. These data show that the genetic diversity of HIV-1 is as high in the general population as in the donor population. This evolution of the genetic diversity of HIV-1 in Gabon requires continuous monitoring of molecular epidemiology. In a transfusion setting, this genetic diversity of HIV being significant can have a negative impact on serological screening tests leading to false negatives. The monitoring of molecular strains in the transfusion environment becomes necessary in order to reduce the transmission of strains. This would strengthen haemovigilance. Phylogenetic analysis of the study strains revealed an evolutionary relationship between the A1, G, F2 subtypes and the recombinants CRF02_AG and CRF45_cpx. Six sequences of study clustered with A1, G, F2 subtypes and recombinants CRF02_AG, CRF45_cpx identified in Gabon, Belgium, in the United States, the Democratic Republic of Congo and Italy. This could be explained by the modification of the molecular map of HIV-1 strains which is due to socio-epidemiological factors, namely the migration and mobility of populations from one country to another and from one continent to another.. In addition, the phylogeny reinforces the argument that the circulation of strains in the sub-region (Central Africa) or the world is very active, favoring several mutations, silent or not. Studies by several authors have shown that these strains (CRFO2_AG, A1, F2, G, CRF45_cpx) are predominant and circulating in the population [43, 47–49]. The data from this study should contribute to improving the screening strategy and epidemiological surveillance of the circulation of HIV-1 strains in donors and also in the general population.

## Conclusion

Viral transmission by blood transfusion persists in blood banks and must be reduced considerably. But it is up to decision-makers in the field of blood transfusion to implement a screening policy centered on nucleic acid research in large blood banks to considerably reduce the residual risk of transmission of viral agents threatening the health of receivers. The current donation screening strategy must be improved by only accepting for distribution blood bags that test negative on the two serological screening tests used (screening test and confirmation test). The use of NAT for screening donations will detect HIV-1 subtypes circulating in donors that are not detectable by serological tests. The genetic diversity of HIV-1 is significant in a transfusion setting with an evolutionary relationship between the different viral strains of the study and reference identified in other countries characterized by the presence of clusters.

## Data Availability

Data generated and analyzed during the current study are not publicly available for confidentiality and data protection reasons but are available from the corresponding author upon reasonable request.

## Abbreviations

Ab: Antibody
Ag: Antigen
CBIRC: Chantal Biya International Reference Center
NCER: National Commitee of Ethics for Research
NBTC: National Blood Transfusion Center
EDTA: Ethylene Diamine Tetra Acetic
ELISA: Enzyme Linked ImmunoSorbent Assay
HIV-1/2: Human Immunodeficiency Virus types 1 and 2
TTI: Transfusion Transmitted Infections
NAT: Nucleic acid test
IR: Incidence Rate
RDT: Rapid Diagnosis Test
RR: Residual Risk
PY: Person -year

## References

[1] Mangala C, Fokam J, Bangola DM, Moundanga M, Nkoa T (2021). Residual risk of HIV in African transfusional setting : systematic review and meta-analysis. International STD Research & Reviews.

[2] Vieira PCM, Lamarao LM, Amaral CEM, Correa ASM, Lima MSM, Barile KAS (2017). Residual risk of transmission of human immunodeficiency virus and hepatitis C virus infections by blood transfusion in northern Brazil. Transfusion.

[3] Awan SA, Junaid A, Sheikh S (2018). Transfusion transmissible infections: maximizing donor surveillance. Cureus.

[4] Peliganga  LB, Mello  VM, de Sousa PSF, Horta  MAP, Soares  ÁD, da Silva Nunes  JP  (2021). Transfusion transmissible infections in blood donors in the Province of Bié, Angola, during a 15-year follow-up, imply the need for pathogen reduction technologies. Pathogens.

[5] Kebede E, Getnet G, Enyew G, Gebretsadik D (2020). Transfusion transmissible infections among voluntary blood donors at dessie blood bank, Northeast Ethiopia: cross-sectional study. Infect Drug Resist.

[6] Steele WR, Dodd RY, Notari EP, Haynes J, Anderson SA, Williams AE (2021). HIV, HCV, and HBV incidence and residual risk in US blood donors before and after implementation of the 12 - month deferral policy for men who have sex with men. Transfusion.

[7] O’Brien  SF , Qi-Long Yi, Wenli  Fan, Scalia  V, Goldman  M, Fearon  MA (2017). Residual risk of HIV, HCV and HBV in Canada. Transfus Apher Sci.

[8] Dodd RY, Crowder LA, Haynes JM, Notari EP, Stramer SL, Steele WR (2020). Screening blood donors for HIV, HCV and HBV at the American red cross: ten-year trends in prevalence, incidence and residual risk, 2007–2016. Transfus Med Rev.

[9] Pillonel J, Pelat C, Tiberghien P, Sauvage C, Danic B, Martinaud C (2020). The evolving blood donor deferral policy for men who have sex with men: impact on the risk of HIV transmission by transfusion in France. Transfusion.

[10] Hassall O, Bates I, M’baya B. Blood transfusion in resource-limited settings. hunter's tropical medicine and emerging infectious diseases (Tenth Edition). 2020 ;19:153–158.

[11] Ware AD, Jacquot C, Tobian AA, Gehrie EA, Ness PM, Bloch EM (2018). Pathogen reduction and blood transfusion safety in Africa: Strengths, limitations and challenges of implementation in low-resource settings. Vox Sang.

[12] Morar MM, Pitman JP, McFarland W, Bloch EM (2016). The contribution of unsafe blood transfusion to human immunodeficiency virus incidence in sub-Saharan Africa: reexamination of the 5% to 10% convention. Transfusion.

[13] Barro L, Drew VJ, Poda GG, Tagny CT, El-Ekiaby M, Owusu-Ofori S (2018). Blood transfusion in sub-Saharan Africa: Understanding the missing gap and responding to present and future challenges. Vox Sang.

[14] Pamatika CM, Parakandji J, Mbeko-Simaleko M, Balekouzou A, Nembi G, Moussa R (2022). Incidence and residual risk of HIV transmission through blood transfusion among regular blood donors in Bangui and Bimbo in the Central African Republic in 2019. Ann Afr Med.

[15] Rerambiah LK, Rerambiah LE, Bengone C, Djoba Siawaya JF (2014). The risk of transfusion-transmitted viral infections at the Gabonese national blood transfusion centre. Blood Transfus.

[16] Eko Mba JM, Ntsame Ndong MJ, Bisseye C (2017). Caractéristiques socio-démographiques associées au risque de transmission du VIH, du VHC et de Treponema pallidum par les donneurs de sang de premier don de Libreville (Gabon): dynamique trisannuelle des infections de 2009 à 2015. International Journal of Biological and Chemical Sciences.

[17] Weimer A, Tagny CT, Tapko JB, Gouws C, Tobian AAR, Ness PM (2018). Blood transfusion safety in sub-Saharan Africa: a literature review of changes and challenges in the 21st century. Transfusion.

[18] Busch MP, Bloch EM, Kleinman S (2019). Prevention of transfusion-transmitted infections. Blood Am Soc Hematol.

[19] Roth WK (2019). History and future of nucleic acid amplification technology blood donor testing. Transfus Med Hemother.

[20] Safic Stanic  H, Babic I, Maslovic M,  Dogic  V, Bingulac-Popovic J, Miletic  M (2017). Three-year experience in NAT screening of blood donors for transfusion transmitted viruses in Croatia. Transfus Med Hemother.

[21] Seed CR, Yang H, Lee JF (2017). Blood safety implications of donors using HIV pre-exposure prophylaxis. Vox Sang.

[22] Makuwa M, Souquière S, Apetrei C, Tevi-Benissan C, Bedjabaga I, Simon F (2000). HIV prevalence and strain diversity in Gabon: the end of a paradox. AIDS [Internet].

[23] Saá P, Townsend RL, Wells P, Janzen MA, Brodsky JP, Stramer SL (2020). Qualification of the Geenius HIV1/2 supplemental assay for use in the HIV blood donation screening algorithm. Transfusion.

[24] Foley BT, Korber BTM, Leitner TK, Apetrei C, Hahn B, Mizrachi I, et al. HIV Sequence Compendium 2018. 2018; Available from: 10.2172/1458915

[25] Kumar S, Stecher G, Li M, Knyaz C, Tamura K. MEGA X: Molecular evolutionary genetics analysis across computing platforms. Battistuzzi FU, editor. Molecular biology and evolution. Oxford University Press (OUP); 2018; 35(6):1547–9. 10.1093/molbev/msy096 PMC5967553 29722887

[26] Mapako T, Janssen MP, Mvere DA, Emmanuel JC, Rusakaniko S, Postma MJ (2016). Impact of using different blood donor subpopulations and models on the estimation of transfusion transmission residual risk of human immunodeficiency virus, hepatitis B virus, and hepatitis C virus in Zimbabwe. Transfusion.

[27] Wongjarupong N,  Oli S, Sanou  M , Djigma F, Kiba Koumare  A, Yonli  AT (2021). Distribution and incidence of blood-borne infection among blood donors from regional transfusion centers in Burkina Faso: a comprehensive study. Am J of Trop Med Hyg.

[28] Arshad A, Borhany M, Anwar N, Naseer I, Ansari R, Boota S (2016). Prevalence of transfusion transmissible infections in blood donors of Pakistan. BMC Hematology.

[29] Ajugwo AO, Erhabor TA, Eledo BO, Eze RI, Digban KA (2017). Prevalence of transfusion transmissible infections in a Nigerian Tertiary Hospital. J Transm Dis Immun.

[30] Ankouane F, Noah Noah D, Atangana MM, Kamgaing Simo R, Guekam PR, Biwolé SM (2016). Séroprévalence des virus des hépatites B et C, du VIH-1/2 et de la syphilis chez les donneurs de sang de l’hôpital central de Yaoundé, région du centre. Cameroun Transfusion Clinique et Biologique.

[31] Camengo Police SM, Bessanguem B, Mofini E, ElowaB, Service G, Guéréndo P, et al. High prevalence of hepatitis B virus infection compared to human immunodeficiency virus among blood donors in Bangui. Open Journal of Gastroenterology. 2020;10:137–143. DOI: 10.4236/ojgas.2020.106014

[32] Slot E, Janssen MP, Marijt-van der Kreek T, Zaaijer HL, Van de Laar TJ (2015). Two decades of risk factors and transfusion-transmissible infections in dutch blood donors. Transfusion.

[33] Candotti D, Sauvage V, Cappy P, Boullahi MA, Bizimana P, Mbensa GO (2020). High rate of hepatitis C virus and human immunodeficiency virus false - positive results in serologic screening in sub - Saharan Africa: adverse impact on the blood supply. Transfusion.

[34] Dadzie I, Muniru S, Adu P, Cudjoe O (2018). Nucleic acid amplification testing detects HIV transmission risk in serologically-tested blood donor units. J Clin Diagn Res.

[35] Yooda AP, Sawadogo S, Soubeiga ST, Obiri-Yeboah D, Nebie K, Ouattara AK (2019). Residual risk of HIV, HCV, and HBV transmission by blood transfusion between 2015 and 2017 at the Regional Blood Transfusion Center of Ouagadougou. Burkina Faso Journal of Blood Medicine.

[36] Dongmo EG, Nsagha DS, Zofou D, Longdoh Njunda A, Nanfack AJ, Fokam J (2020). Residual risk of HIV transmission through blood transfusion in five blood banks in Cameroon. The Journal of Medical Research.

[37] Aboud S, Nyale E, Mosha F (2011). Acute HIV-1 Infection in Antigen/Antibody-negative Blood Donors in Dar es Salaam, Tanzania. Tanzan J Health Res.

[38] Yooda AP, Soubeiga ST, Yacouba Nebie K, Diarra B, Sawadogo S, Ouattara AK (2018). Impact of multiplex PCR in reducing the risk of residual transfusion-transmitted human immunodeficiency and hepatitis B and C viruses in Burkina Faso. Mediterr J Hematol Infect Dis.

[39] Mathur A, Dontula S, Jagannathan L (2017). A study of centralized individual donor nucleic acid testing for transfusion transmitted infections to improve blood safety in Karnataka. India Glob J Transfus Med.

[40] Pawar A, Jagani R, Dimri U, Kumar S. Experience of individual donor nucleic acid testing on screening of blood donors for human immunodeficiency virus, Hepatitis C Virus, and Hepatitis B Virus at an Apex blood bank of Northern India. Medical Journal of Dr DY Patil Vidyapeeth [Internet]. Medknow. 2021.

[41] Aramani SS, Bommanahalli  BP, Kammar  SM (2019). Efficacy of nucleic acid amplification Test (NAT) over enzyme linked immuno sorbent assay (ELISA) in Detecting Transfusion Transmissible Infections (TTI) among blood donors at a tertiary care centre. Ann Pathol Lab Med.

[42] Obeng BM, Bonney EY, Asamoah-Akuoko L, Nii-Trebi NI, Mawuli G, Abana CZY (2020). Transmitted drug resistance mutations and subtype diversity amongst HIV-1 sero-positive voluntary blood donors in Accra. Ghana Virol J.

[43] Olusola BA, Olaleye DO, Odaibo GN (2021). New infections and HIV - 1 subtypes among febrile persons and blood donors in Oyo State. Nigeria Journal of Medical Virology.

[44] Tagny CT, Mbanya D, Leballais L, Murphy E, Lefrère JJ, Laperche S (2011). Reduction of the risk of transfusion-transmitted human immunodeficiency virus (HIV) infection by using an HIV antigen/antibody combination assay in blood donation screening in Cameroon. Transfusion.

[45] Vubil A, Mabunda N, Ismael N, Ramalho D, Morgado MG, Couto-Fernandez JC (2016). Genetic diversity and transmitted drug resistance of HIV-1 Subtypes in blood donors candidates in Northern Mozambique. J AIDS Clin Res.

[46] Caron M, Lekana-Douki SE, Makuwa M, Obiang-Ndong GP, Biba O, Nkoghé D (2012). Prevalence, genetic diversity and antiretroviral drugs resistance-associated mutations among untreated HIV-1-infected pregnant women in Gabon, central Africa. BMC Infect Dis.

[47] Teto G, Tagny CT, Mbanya D, Fonsah JY, Fokam J, Nchindap E (2017). Gag P2/NC and pol genetic diversity, polymorphism, and drug resistance mutations in HIV-1 CRF02_AG- and non-CRF02_AG-infected patients in Yaoundé, Cameroon. Sci Rep.

[48] Chow WZ, Bon AH, Keating S, Anderios F, Halim HA, Takebe Y, et al. Extensive Genetic Diversity of HIV-1 in incident and prevalent infections among Malaysian blood donors: multiple introductions of HIV-1 genotypes from highly prevalent countries. Paraskevis D, editor. PLOS ONE. Public Library of Science (PLoS); 2016;11(8):e0161853. 10.1371/journal.pone.0161853 PMC5004849 27575746

[49] Bes M, Piron M, Casamitjana N, Gregori J, Esteban JI, Ribera E (2017). Epidemiological trends of HIV-1 infection in blood donors from Catalonia, Spain (2005–2014). Transfusion.

